# Serine-dependent redox homeostasis regulates glioblastoma cell survival

**DOI:** 10.1038/s41416-020-0794-x

**Published:** 2020-03-17

**Authors:** Anna L. Engel, Nadja I. Lorenz, Kevin Klann, Christian Münch, Cornelia Depner, Joachim P. Steinbach, Michael W. Ronellenfitsch, Anna-Luisa Luger

**Affiliations:** 1 Dr. Senckenberg Institute of Neurooncology, University Hospital Frankfurt, Goethe University, Frankfurt am Main, Germany; 2 University Cancer Center Frankfurt (UCT), University Hospital Frankfurt, Goethe University, Frankfurt am Main, Germany; 3 German Cancer Consortium (DKTK), Partner Site Frankfurt/Mainz, Frankfurt am Main, Germany; 4 Frankfurt Cancer Institute (FCI), Frankfurt am Main, Germany; 5 Institute of Biochemistry II, Goethe University, Frankfurt am Main, Germany; 6 Cardio-Pulmonary Institute, Frankfurt am Main, Germany; 7 Institute of Neuropathology, University of Giessen, Giessen, Germany

**Keywords:** CNS cancer, Mechanisms of disease, Molecular medicine, CNS cancer

## Abstract

**Background:**

The amino acid serine is an important substrate for biosynthesis and redox homeostasis. We investigated whether glioblastoma (GBM) cells are dependent on serine for survival under conditions of the tumour microenvironment.

**Methods:**

Serine availability in GBM cells was modulated pharmacologically, genetically and by adjusting serine and glycine concentrations in the culture medium. Cells were investigated for regulation of serine metabolism, proliferation, sensitivity to hypoxia-induced cell death and redox homeostasis.

**Results:**

Hypoxia-induced expression of *phosphoglycerate dehydrogenase (PHGDH)* and the mitochondrial *serine hydroxymethyltransferase (SHMT2)* was observed in three of five tested glioma cell lines. Nuclear factor erythroid 2-related factor (Nrf) 2 activation also induced *PHGDH* and *SHMT2* expression in GBM cells. Low levels of endogenous PHGDH as well as *PHGDH* gene suppression resulted in serine dependency for cell growth. Pharmacological inhibition of PHGDH with CBR-5884 reduced proliferation and sensitised cells profoundly to hypoxia-induced cell death. This effect was accompanied by an increase in reactive oxygen species and a decrease in the NADPH/NADP^+^ ratio. Similarly, hypoxia-induced cell death was enhanced by *PHGDH* gene suppression and reduced by *PHGDH* overexpression.

**Conclusions:**

Serine facilitates adaptation of GBM cells to conditions of the tumour microenvironment and its metabolism could be a plausible therapeutic target.

## Background

Glioblastoma (GBM) is the most common primary CNS malignancy in adults with a dismal prognosis.^1^ Multimodal therapeutic approaches including tumour resection, radiochemotherapy and tumour treating fields yield overall survival times of only about 21 months^2,3^ and new treatment approaches are urgently needed.

Hypoxia is a common feature of the microenvironment of GBMs^4–7^ caused by an imbalance of tumour growth and vascular supply.^8,9^ Reactive oxygen species (ROS) are common by-products of aerobic metabolism primarily originating from mitochondria due to an incomplete reduction of oxygen in the electron transport chain.^10^ ROS levels can be enhanced by elevated metabolic activity, hypoxia or chemotherapy.^11–13^ Excessive ROS production can lead to the depletion of reducing substrates, ultimately resulting in oxidative damage and cytotoxicity.^14^

Reprogramming energy metabolism has more recently been acknowledged as a hallmark of cancer.^15^ One well-known and extensively studied phenomenon of an altered cancer metabolism is aerobic glycolysis or the so-called Warburg effect. This describes the preferentially glucose metabolism via glycolysis without subsequent entry of substrates into the citric acid cycle despite the availability of oxygen resulting in a potential waste of energy.^16^ Another metabolic pathway that has recently attracted attention in cancers is the serine synthesis pathway (SSP). In certain cancer types, such as breast cancer, melanoma and non-small cell lung cancer, the central enzymes of serine metabolism are upregulated and high SSP activity defines a more aggressive tumour subtype with worse prognosis.^17,18^

Besides its function as a synthesis substrate for proteins and lipids,^19,20^ serine contributes to the one-carbon pool (1CM) to promote nucleotide synthesis.^19^ Additionally, 1CM is a source for NADPH production via methylenetetrahydrofolate dehydrogenase (MTHFD), which catalyses the conversion of methylenetetrahydrofolate (MTHF) and NADP^+^ to formyl-tetrahydrofolate (formyl-THF) and NADPH.^21^ NADPH increases the cellular antioxidative capacity by regenerating the cellular pool of reduced glutathione and thioredoxin.^22^

Apart from its import via amino acid transporters^23,24^ serine can be synthesised de novo from the glycolytic intermediate 3-phosphoglycerate (3-PG).^25^ This first and rate limiting step to divert substrates from glycolysis to serine synthesis is catalysed by 3-PG dehydrogenase (PHGDH).^26^ Focal amplifications of the *PHGDH* gene have been described in breast cancer and melanoma.^18,27^ Recently, it has been demonstrated for these two entities that expression of PHGDH is a relevant factor for tumour cell proliferation when serine supply is limited.^28^ In gliomas, PHGDH expression increases with WHO grade and silencing of *PHGDH* leads to reduced GBM cell proliferation and invasion.^29^ Lately, a novel selective small-molecule inhibitor of PHGDH, CBR-5884, has been identified.^30^ Serine hydroxymethyltransferases (SHMTs) catalyse the conversion of serine to glycine and vice versa.^31^ SHMT1, the cytoplasmatic isoform, does not significantly contribute to the production of glycine, whereas SHMT2, the mitochondrial isoform, is an important source of glycine in proliferating cells.^32,33^ In GBM, pseudopallisading cells surrounding necrotic regions express high levels of SHMT2 and glycine decarboxylase (GLDC). In those cells, SHMT2 reduces oxygen consumption to adapt to microenvironmental conditions.^34^

In this project we modulated serine availability and SSP enzymatic activity under conditions mirroring the GBM microenvironment. Serine- and glycine-free culture medium as well as CBR-5884 were used to limit import as well as endogenous serine production. We analysed a panel of glioma cell lines for basal expression of key SSP enzymes, propensity to serine synthesis and dependence on exogenous serine supplementation. Furthermore, expression of SSP enzymes, as well as cell survival, oxidative stress and NADPH production, were investigated under starvation conditions. We report that inhibition of SSP activity sensitises GBM cells to hypoxia-induced cell death by increasing reactive oxygen species.

## Methods

### Reagents, cell lines and culture conditions

All reagents not specified were purchased from Sigma (St. Louis, MO, USA). CBR-5884, a PHGDH inhibitor, and RA 839, a Nrf2 (nuclear factor erythroid 2-related factor) activator, were purchased from Tocris (Bristol, UK). LNT-229, LN-308, LN-428 and G55 cells have been described.^35^ LNT-229 and LN-308 cells were a kind gift from N. de Tribolet (Lausanne, Switzerland), G55 cells were a kind gift from Manfred Westphal and Kathrin Lamszus (Hamburg), LN-428 cells and LN-464 cells were a kind gift from Monika Hegi (Lausanne). MDA-MB-231 and MDA-MB-464 cells were a kind gift from Winfried Wels (Frankfurt, Germany).

Wildtype cell lines were maintained as described.^35^ For experiments glycine- and serine-free DMEM (US Biological Life Sciences, catalog no. D9800-03/D9802-01, Salem, MA, USA) was supplemented with glucose or serine as indicated. FCS included serine and its supplementation yielded serine concentrations of 20 µM (in comparison to 400 µM when serine was replenished). pLKO.1- and pTetOne-transfected cells were maintained in medium with 2 µg/ml puromycin. 0.1 µg/ml doxycycline was added to induce gene expression of PHGDH from pTetOne-transfected cells. To compare sub cell lines equal cell densities were confirmed by crystal violet (CV) staining as described.^36^

### Generation of PHGDH gene suppressed and PHGDH overexpressing cells

The pLKO.1 plasmid (Sigma, Clone-ID: TRCN 00000 28532) was used to mediate stable shRNA-mediated gene suppression of *PHGDH*. Control cells were transfected with a pLKO.1 plasmid with a non-targeting shRNA sequence (Addgene, catalog no. 1864, Watertown, MA, USA). Attractene (Qiagen, Hilden, Germany) was used for transfection. LNT-229 pTetOne PHGDH cells have been described.^37^ Expression of *PHGDH* in the cell pool and single cell clones was quantified by qPCR after incubation with or without doxycycline. For further analysis one single cell clone with a 14-fold higher expression of *PHGDH* compared to control was used.

### Induction of hypoxia

Hypoxia was induced as previously described.^36,38,39^ Briefly, 0.1% oxygen was induced by incubation in GasPak™ pouches for anaerobic culture (Becton-Dickinson, Heidelberg, Germany).^36,38^ Oxygen deprivation of 1% as well as 5% oxygen was induced in a Labotect incubator (Goettingen, Germany) as described.^39^

### RNA extraction and quantitative reverse transcription-PCR (qRT-PCR) analysis

The qPCR protocol employed has already been described.^35^ Primer pairs are listed in the supplement (Suppl. Table 1). 18S and *SDHA* were both used as housekeeping genes for normalisation.

### Immunoblot analysis

Immunoblot was performed as recently described.^35^ Membranes were probed with antibodies to PHGDH (Santa Cruz Biotechnology, Dallas, TX, USA), SHMT1, SHMT2 (Atlas Antibodies, Bromma, Sweden) or actin (Santa Cruz Biotechnology, Dallas, TX, USA). The secondary anti-mouse and anti-goat antibodies were purchased from Santa Cruz Biotechnology (Dallas, TX, USA). The secondary anti-rabbit antibody was purchased from Jackson ImmunoResearch (Cambridgeshire, UK).

### Amino acid measurements by LC-MS/MS

Amino acid measurements by LC-MS/MS was performed as recently described.^40^ A detailed protocol is included in the supplement (Supplementary Methods).

### Cell density and cell viability assays

Cell density measurement by crystal violet (CV) staining as well as cell viability measurement by lactate dehydrogenase (LDH) release assay with the Cytotoxicity Detection Kit (LDH) (Roche, Mannheim, Germany) have already been described.^35^

### Reactive oxygen species measurement

Reactive oxygen species analysis was also performed as described previously.^35^

### NADPH/NADP^+^ measurement

NADPH and NADP^+^ were measured with a luminescence-based assay (NADP/NADPH-Glo assay kit, Promega, Madison, WI, USA) according to the manufacturer’s protocol.

### Statistical analysis

Quantitative data are expressed as indicated including standard deviation (S.D.). *P*-values were derived from two-tailed student’s *t*-tests. Values of *P* > 0.05 were considered not significant (n.s.). Values of *P* < 0.05 and *P* < 0.01 were considered significant and highly significant (Excel, Microsoft, Seattle, WA, USA).

## Results

### Expression of key enzymes of serine metabolism varies between different glioma cell lines

**Fig. 1:**
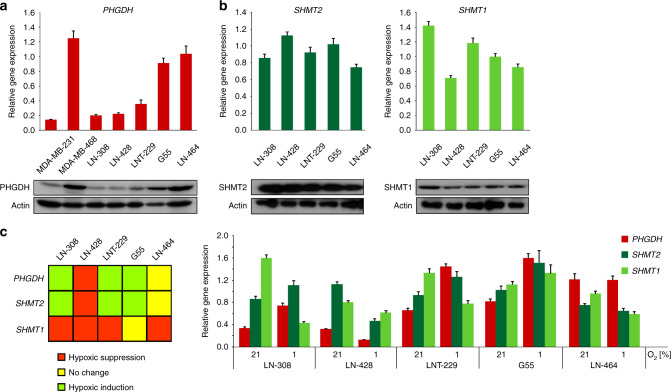
Expression of key enzymes of SSP under normoxic and hypoxic conditions. **a**–**b** Gene expression (upper panel) and protein levels (lower panel) of the SSP enzymes PHGDH (**a**), SHMT1 and 2 (**b**) in breast cancer (MDA-MB-231 and MDA-MB-468) and glioma (LN-308, LN-428, LNT-229, G55 and LN-464) cell lines were investigated under standard conditions (DMEM containing 10% FCS and 25 mM glucose under normoxia) by qPCR and immunoblot. Values are normalised to *18* *S* as well as *SDHA* housekeeping gene expression (*n* = 3, mean ± SD). Cellular lysates were analysed by immunoblot with antibodies for PHGDH, SHMT1, SHMT2 and actin. **c** Gene expression of the SSP enzymes *PHGDH*, *SHMT1* and *2* in glioma cell lines were investigated under starvation conditions (24 h in serum-free medium and 1% oxygen (O_2_)). Gene expression was measured by qPCR. Values are normalised to *18* *S* as well as *SDHA* housekeeping gene expression (*n* = 3, mean ± SD). Significant gene induction (*p < 0.05 or **p < 0.01) is illustrated by green boxes, significant gene suppression (*p < 0.05 or **p < 0.01) is illustrated by red boxes and no significant change in gene expression is illustrated by yellow boxes.

mRNA expression and protein levels of key enzymes of serine metabolism were investigated in a panel of glioma cell lines. Breast cancer cell lines with reportedly low (MDA-MB-231) and high (MDA-MB-468) PHGDH expression^18^ were analysed for comparison (Fig. 1a). GBM cell lines displayed a broad spectrum of PHGDH expression: LN-308 and LN-428 showed low expression comparable to PHGDH expression in MDA-MB-231 cells (Fig. 1a). In contrast G55 and LN-464 had almost similar PHGDH expression levels as MDA-MB-468 (Fig. 1a). LNT-229 had intermediate PHGDH levels (Fig. 1a). SHMT1 and 2 expression levels varied only moderately between the tested glioma cell lines (Fig. 1b).

### *PHGDH* and *SHMT2* but not *SHMT1* are upregulated under hypoxic conditions in LN-308, LNT-229 and G55 cells

An upregulation of SSP enzymes in hypoxia has previously been found in breast cancer cells.^41^ In line with that, hypoxia led to an upregulation of *SHMT2* and *PHGDH* expression in MDA-MB-231 und MDA-MB-468 cells while *SHMT1* expression was not induced or even reduced under hypoxia (Suppl. Fig. 1A). To investigate a potential adaption of GBM cell SSP under deprivation conditions, expression levels of *PHGDH*, *SHMT1* and *2* were tested under hypoxic conditions with 1% hypoxia as already tested for breast cancer cell lines^41^ (Fig. 1c). An upregulation of *PHGDH* and *SHMT2* was also observed in LN-308, LNT-229 and G55 cells. However, *PHGDH* and *SHMT2* levels were unchanged or reduced in LN-464 or LN-428 cells. *SHMT1* levels were unchanged or reduced in all tested cell lines.

As areas of GBMs can exhibit levels of profound hypoxia as low as 0.1% oxygen^42^ and distinct areas of solid tumours display oxygen concentrations between 5% oxygen^43–47^ and 0.1% oxygen,^48,49^ we also investigated gene expression of SSP enzymes under 0.1 and 5% oxygen (Suppl. Fig. 1B). An upregulation of *PHGDH* was also observed in LN-308 and LNT-229 cells under 0.1% oxygen. *SHMT2* was upregulated in LN-308 and G55 cells under 0.1% oxygen. *SHMT1* gene expression was suppressed in all tested cell lines, except LN-308 under 0.1% oxygen. Under 5% oxygen only a slight induction of *SHMT2* was observed in LN-308 and LNT-229 cells. *PHGDH* gene expression was not induced under 5% oxygen in all tested cell lines. On the contrary, LN-428 and LN-464 even showed a gene suppression of *PHGDH* under 5% oxygen. In contrast to the published results of breast cancer cells^41^ the hypoxia-induced upregulation of *SHMT2* was only affected by a double gene-suppression of HIF-1α and HIF-2α in GBM cells whereas a single HIF-1α or HIF-2α gene-suppression as well as a double gene-suppression of HIF-1α and HIF-2α did not prevent induction of *PHGDH* under hypoxia (Suppl. Fig. 2A). *SHMT1* expression was unaffected by hypoxia (Suppl. Fig. 2A). A regulation of *PHGDH* and *SHMT2* by the transcription factor Nrf2 has recently been reported in non-small cell lung cancer cells.^17^ Similarly, we found an induction of *PHGDH* and *SHMT2* but not *SHMT1* by the Nrf2 activator RA 839 in LNT-229 and G55 cells (Suppl. Fig. 2B). Expression levels of the Nrf2-targets *heme oxygenase 1 (HO-1)* and *thioredoxin 1 (TXN-1)* are shown as indicators for Nrf2 activation by RA 839 (Suppl. Fig. 2B).

### Serine availability is required for tumour cell growth

**Fig. 2:**
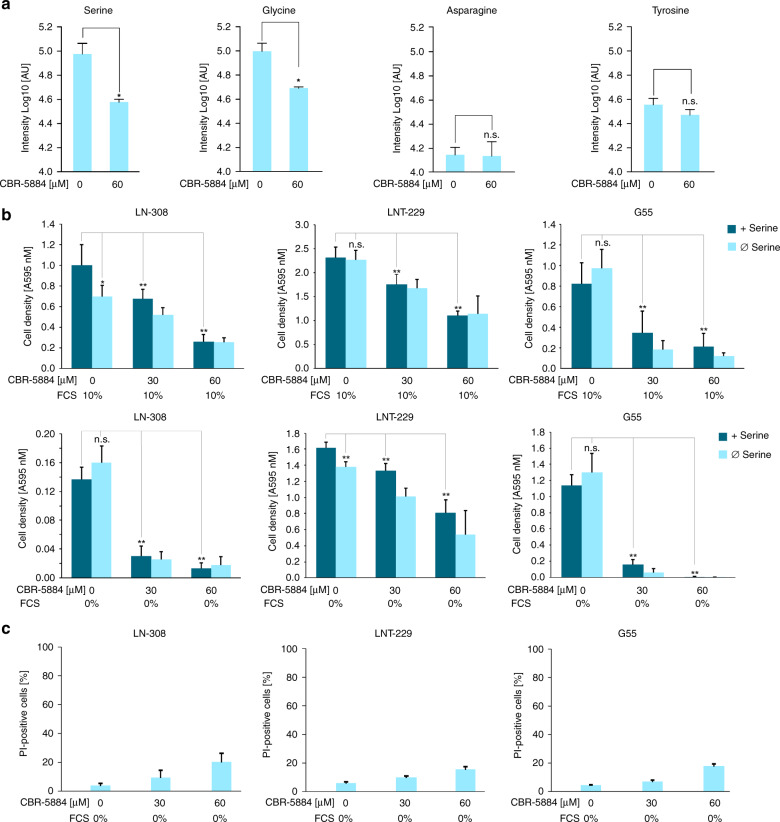
Effects of serine availability on tumour cell growth. **a** G55 cells were incubated in serum-free medium without glucose restriction (25 mM) depleted for glycine and serine with vehicle or 60 µM CBR-5884 as indicated for 24 h. Serine, glycine, asparagine and tyrosine levels were measured by LC-MS/MS (*n* = 3, mean ± SD, n.s. not significant, *p < 0.05). **b** LN-308, LNT-229 and G55 cells were incubated in DMEM (25 mM glucose) depleted for glycine and serine with or without CBR-5884 as indicated. Serine was replenished as indicated, with (upper panel) and without (lower panel) 10% FCS. Cells were incubated for 4 days. Cell density was measured by crystal violet staining at the beginning of cultivation and at 4 days (*n* = 6, mean ± SD, n.s. not significant, *p < 0.05, **p < 0.01). C, LN-308, LNT-229 and G55 cells were incubated in serum-free DMEM depleted for glycine and serine with or without CBR-5884 as indicated. Cell death was quantified by PI-uptake after 4 days (*n* = 3, mean ± SD).

To investigate the effect of serine deprivation on tumour cell proliferation we targeted three potential sources of serine: (i) serine import, (ii) serine synthesis from glycine *via* SHMT1 and 2 and (iii) de novo synthesis via PHGDH by employing glycine- and serine-free DMEM as well as the novel small-molecule PHGDH inhibitor CBR-5884.^30^ The combination of glycine- and serine-free DMEM with 60 µM CBR-5884 resulted in a significantly reduced intracellular serine and glycine level in comparison to vehicle in G55 cells (Fig. 2a). In contrast, asparagine and tyrosine (used as a control) levels were not affected by CBR-5884. Under serum containing culture conditions serine and glycine deprivation alone led to a significant decrease of tumour cell proliferation in LN-308 cells with an intrinsically low PHGDH expression. Proliferation of cell lines with intermediate (LNT-229) or high (G55) PHGDH expression was not affected by serine and glycine restriction under serum containing conditions (Fig. 2b, upper panel). Under serum-free conditions cell proliferation was inhibited in LNT-229 cells by serine and glycine deprivation but not in G55 cells. LN-308 cells showed almost no proliferation under serum-free conditions (Fig. 2b, lower panel). PHGDH inhibition with CBR-5884 inhibited tumour cell growth in all tested cell lines in a dose-dependent manner. This effect was even stronger under serum-free culture conditions (Fig. 2b, lower panel). In a parallel PI-FACS analysis toxicity of CBR-5884 under serum-free, serine- and glycine-free conditions was analysed and revealed only mild to moderate cell death under increasing CBR-5884 concentrations (Fig. 2c).

### Serine restriction sensitises for hypoxia-induced cell death and impairs redox homeostasis

**Fig. 3:**
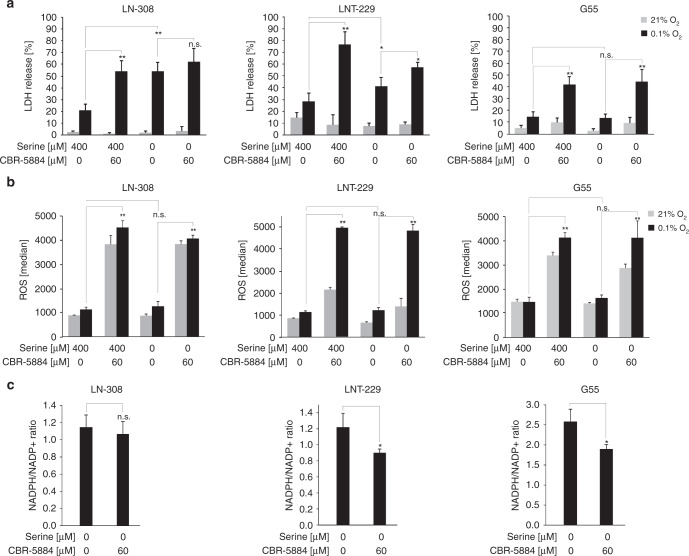
Serine restriction sensitises for hypoxia-induced cell death and increases intracellular ROS levels. **a**–**c** LN-308, LNT-229 and G55 cells were exposed to glucose restricted (2 mM glucose) serum- and glycine-free DMEM under normoxic or hypoxic (0.1% oxygen (O_2_)) conditions with or without CBR-5884 as indicated. Serine was replenished as indicated. **a** Cell death was quantified by LDH-release (*n* = 4, mean ± S.D., n.s. not significant, *p < 0.05, **p < 0.01). **b** Reactive oxygen species (ROS) levels were measured by H2DCFDA-FACS (*n* = 3, mean ± SD, n.s. not significant, **p < 0.01). **c** Analysis of NADPH/NADP^+^ ratios was performed by a luminescence-based assay (*n* = 3, mean ± SD, n.s. not significant, *p < 0.05).

We next investigated whether intracellular serine levels influence tumour cell survival under conditions of the tumour microenvironment. LN-308 (low PHGDH expression) and LNT-229 (moderate PHGDH expression) showed increased cell death under serine and glycine deprivation alone (Fig. 3a). G55 (high PHGDH expression) cells were unaffected by serine and glycine deprivation. CBR-5884 increased hypoxia-induced cell death in all tested cell lines. Similar results were obtained by PI-FACS analysis (data not shown). In line with these results ROS levels were increased under treatment with CBR-5884 in all tested cell lines (Fig. 3b). Measurement of the NADPH/NADP^+^ ratio showed that PHGDH inhibition led to a significant decrease of the NADPH/NADP^+^ ratio in cells with moderate (LNT-229) or high (G55) PHGDH expression (Fig. 3c). However, in LN-308 cells (low PHGDH expression) only a trend towards a lower NADPH/NADP^+^ ratio could be observed.

### PHGDH gene suppression sensitises human GBM cells to hypoxia-induced cell death

**Fig. 4:**
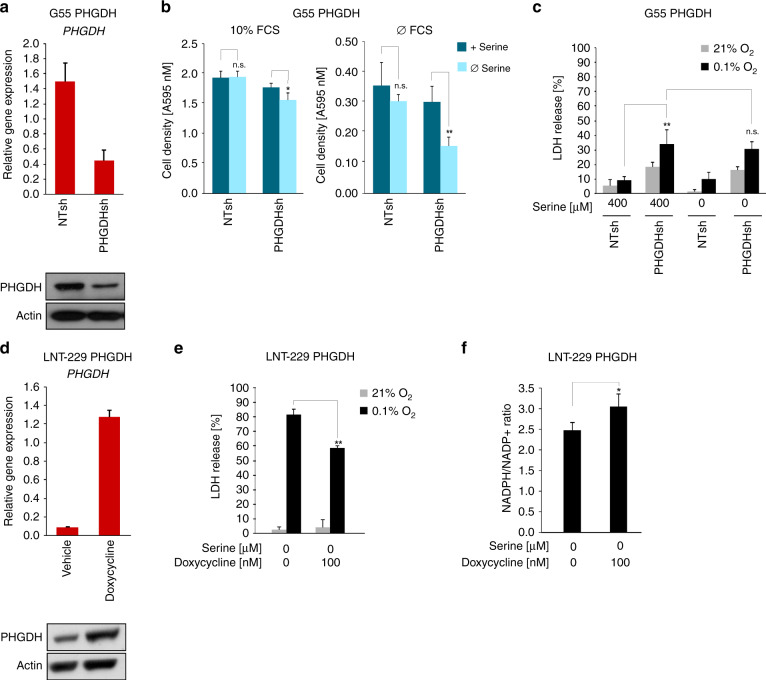
*PHGDH* gene suppression sensitises while PHGDH overexpression protects human GBM cells from hypoxia-induced cell death. **a** G55 PHGDHsh and control cells (non-targeting sequence, NTsh) were analysed by qPCR and immunoblot. *PHGDH* gene suppression was confirmed. qPCR values are normalised to *18* *S* as well as *SDHA* housekeeping gene expression (*n* = 3, mean ± SD). **b** G55 PHGDHsh and control cells were incubated in DMEM with or without 10% FCS as indicated. Medium was depleted for glycine and serine and serine was replenished where indicated. Cell density was measured by crystal violet staining at the beginning of cultivation and at 4 days (*n* = 6, mean ± S.D., n.s. not significant, *p < 0.05, **p < 0.01). **c** G55 PHGDHsh and control cells were exposed to glucose restricted (2 mM glucose) serum-free DMEM under normoxic or hypoxic (0.1% oxygen (O_2_)) conditions. Serine was replenished as indicated. Cell death was quantified by LDH release (*n* = 4, mean ± S.D., n.s. not significant, **p < 0.01). **d** LNT-229 pTetOne PHGDH cells were cultured with vehicle or 0.1 µg/mL doxycycline for 24 h. *PHGDH* gene induction was confirmed by qPCR and immunoblot. qPCR values are normalised to *18* *S* as well as *SDHA* housekeeping gene expression (*n* = 3, mean ± SD). E-F, LNT-229 pTetOne PHGDH cells were preincubated with or without 0.1 µg/ml doxycycline for 24 h and exposed to glucose restricted (2 mM glucose) serum- and serine-free DMEM with or without 0.1 µg/ml doxycycline under normoxic or hypoxic (0.1% oxygen (O_2_)) conditions. **e** Cell death was quantified by LDH release (*n* = 4, mean ± S.D., **p < 0.01). (*n* = 4, mean ± S.D.) **f** Analysis of NADPH/NADP^+^ ratios were performed by a luminescence-based assay (*n* = 3, mean ± SD, *p < 0.05).

To further confirm the robustness of the observed phenotype and rule out off-target effects of CBR-5584, cells with gene suppression of *PHGDH* were generated. G55 cells were chosen due to their high endogenous PHGDH level. QPCR and immunoblot confirmed stable gene suppression of *PHGDH* (PHGDHsh) compared to control cells (NTsh) (Fig. 4a). Cell proliferation was impaired by serine and glycine withdrawal under serum-free and serum containing culture conditions only in G55 PHGDHsh cells mimicking the phenotype of cells with low PHGDH expression (Fig. 4b). Furthermore, G55 PHGDHsh cells displayed enhanced sensitivity to hypoxia-induced cell death coherent with results obtained with pharmacological PHGDH inhibition (Fig. 4c). However, serine withdrawal did not affect sensitivity to hypoxia-induced cell death in PHGDHsh cells (Fig. 4c).

### PHGDH overexpression protects human GBM cells from hypoxia-induced cell death

LNT-229 cells that inducibly overexpress *PHGDH* were generated (LNT-229 pTetOne PHGDH). Gene induction by doxycycline was confirmed by qPCR and immunoblot (Fig. 4d). In contrast to PHGDHsh cells, LNT-229 pTetOne PHGDH cells displayed protection from hypoxia-induced cell death when *PHGDH* was induced (Fig. 4e). In addition, *PHGDH* induction increased the NADPH/NADP^+^ ratio (Fig. 4f).

## Discussion

Our results describe serine metabolism as an important regulator of cellular redox homeostasis and tumour cell survival under conditions of the glioma microenvironment.

The relevance of serine metabolism in GBM cells was first shown by demonstrating an induction of enzymes of SSP under hypoxic conditions that mirror the in vivo GBM situation. Our results on hypoxic upregulation of *PHGDH* and mitochondrial *SHMT2* (Fig. 1c, Suppl. Fig. 1B) emphasise the robustness of the phenomenon of a hypoxia dependent upregulation of SSP enzymes in cancer.^34,41^ Furthermore, we could show that overexpression of PHGDH protected GBM cells from hypoxia-induced cell death (Fig. 4e) and sustained NADPH/NADP^+^ ratios under starvation conditions (Fig. 4f). Our results affirm that high PHGDH levels protect GBM cells under most adverse conditions of the glioma microenvironment by maintaining redox homeostasis. Therefore, induction of SSP enzymes seems to serve as an adaptive response to adverse conditions of the tumour microenvironment. Vice versa, we could show for the first time that PHGDH inhibition with the new small-molecule inhibitor CBR-5884 sensitises GBM cells towards the conditions of the microenvironment, including glucose deprivation and severe hypoxia (Fig. 3a, Suppl. Fig. 3). This effect could be observed in all tested cell lines regardless of the PHGDH expression levels. Hypoxia-induced cell death under PHGDH inhibition was accompanied by an increase in intracellular ROS (Fig. 3b, Suppl. Fig. 3). Furthermore, for LNT-229 and G55 cells with moderate to high PHGDH expression levels, a decrease in the NADPH/NADP^+^-ratio could also be observed after treatment with CBR-5884 (Fig. 3c, Suppl. Fig. 3).

Beyond that, PHGDH inhibition with CBR-5884 reduced cell proliferation in a dose-dependent manner in all tested cell lines regardless of the PHGDH expression levels (Fig. 2b). These results are in contrast to the data of the inhibitor’s developers, who observed only growth inhibitory effects of CBR-5884 in cancer cell lines with high PHGDH expression levels and a high propensity for serine synthesis.^30^ However, in this publication only concentrations up to 30 µM CBR-5884 were applied for cell proliferation assays, which is below the IC_50_ of 33 (±12) µM.^30^

Remarkably, GBM cells varied in their dependency on serine import. Serine deprivation alone showed only a significant reduction of cell growth and an increase in hypoxia-induced cell death in cells with low or moderate PHGDH expression (LN-308 and LNT-229) suggesting their dependency on import of extracellular serine (Figs. 2b, 3a). In contrast, G55 with higher PHGDH expression levels were less susceptible to sole serine starvation (Figs. 2b, 3a). Our results on growth propensity to serine import are in line with recent findings demonstrating that either increased PHGDH expression or increased serine supply provide a proliferative advantage in breast cancer and melanoma.^28^ Corroborating these results, gene suppression of *PHGDH* rendered G55 cells susceptible to serine starvation under normoxia (Fig. 4b). In contrast, however, CBR-5884-mediated PHGDH inhibition did not influence the growth propensity of G55 cells to serine import (Fig. 2b). Furthermore, treatment with CBR-5884 as well as gene suppression of *PHGDH* in G55 did not lead to a sensitisation to serine withdrawal under hypoxic conditions (Figs. 3a, 4c). One reason for this discrepancy could be a potential contribution of other SSP enzymes or factors so that PHGDH inhibition alone is not sufficient to sensitise to serine withdrawal. Also, PHGDH inhibition could already induce a strong growth inhibition and sensitisation to hypoxia-induced cell death in G55 cells narrowing the potential of serine depletion for additional effects.

Taken together high intracellular serine levels seem to be favourable for tumour cells under stressful conditions of the glioma microenvironment. Both proliferation and survival were impaired by serine pathway inhibition under nutrient and oxygen deprivation. As serine and glycine are nonessential amino acids, one possible and feasible therapeutic approach could be a serine/glycine-free diet. In line with that, mice bearing colorectal xenograft tumours fed with a diet lacking serine and glycine displayed a reduction in tumour volume and a prolonged survival.^50^ Moreover, several in vivo mouse experiments with PHGDH inhibitors alone or as a combined approach have shown positive antitumour effects in different tumour entities, such as breast cancer, renal cell carcinoma and hepatocellular carcinoma.^51–53^ Our data indicate that a combination of a serine/glycine-free diet with PHGDH inhibition could be an even more effective approach. Besides inhibitors of PHGDH, recently inhibitors of SHMT1/2 have been described.^54^ Serine depletion by diet and/or PHGDH inhibition might cause severe neurological side effects. In this respect *PHGDH* conditional knockout mice show mild microcephaly and forebrain atrophy.^55^ However, effects of congenital gene depletion cannot be transferred to the setting of a mature human brain. Furthermore, in our settings PHGDH inhibition with CBR-5884 seemed to allow residual enzyme activity (Fig. 2a) while still impacting cell survival (Fig. 3a). Encouragingly, previous in vivo mouse experiments with PHGDH inhibitors demonstrated a tolerable toxicity profile with neither weight loss nor abnormal behaviour.^51–53^ Therefore, further in vivo animal studies on neurological side effects as well as clinical trials on the efficacy and safety of the combination of serine/glycine deprivation with inhibition of the SSP, especially of PHGDH, are exciting future options for a serine-targeted therapeutic approach in cancer.

## Supplementary information

Supplemental Material

## Data Availability

The datasets used and/or analysed during the current study are available from the corresponding author on reasonable request. Supplementary information is available at the British Journal of Cancer’s website.
