# Correction: Serine-dependent redox homeostasis regulates glioblastoma cell survival

**DOI:** 10.1038/s41416-021-01517-4

**Published:** 2021-08-06

**Authors:** Anna L. Engel, Nadja I. Lorenz, Kevin Klann, Christian Münch, Cornelia Depner, Joachim P. Steinbach, Michael W. Ronellenfitsch, Anna-Luisa Luger

**Affiliations:** 1 Dr. Senckenberg Institute of Neurooncology, University Hospital Frankfurt, Goethe University, Frankfurt am Main, Germany; 2 University Cancer Center Frankfurt (UCT), University Hospital Frankfurt, Goethe University, Frankfurt am Main, Germany; 3 German Cancer Consortium (DKTK), Partner Site Frankfurt/Mainz, Frankfurt am Main, Germany; 4 Frankfurt Cancer Institute (FCI), Frankfurt am Main, Germany; 5 Institute of Biochemistry II, Goethe University, Frankfurt am Main, Germany; 6 Cardio-Pulmonary Institute, Frankfurt am Main, Germany; 7 Institute of Neuropathology, University of Giessen, Giessen, Germany

**Keywords:** CNS cancer, Mechanisms of disease, Molecular medicine, CNS cancer

Correction to: *British Journal of Cancer* 10.1038/s41416-020-0794-x, published online 17 March 2020

The article “Serine-dependent redox homeostasis regulates glioblastoma cell survival”, written by Anna L. Engel, Nadja I. Lorenz, Kevin Klann, Christian Münch, Cornelia Depner, Joachim P. Steinbach, Michael W. Ronellenfitsch and Anna-Luisa Luger, was originally published electronically on the publisher’s internet portal on 17 March 2020 without open access. With the author(s)’ decision to opt for Open Choice the copyright of the article changed on 23 July 2021 to © The Author(s) 2021 and the article is forthwith distributed under a Creative Commons Attribution 4.0 International License, which permits use, sharing, adaptation, distribution and reproduction in any medium or format, as long as you give appropriate credit to the original author(s) and the source, provide a link to the Creative Commons licence, and indicate if changes were made. The images or other third party material in this article are included in the article’s Creative Commons licence, unless indicated otherwise in a credit line to the material. If material is not included in the article’s Creative Commons licence and your intended use is not permitted by statutory regulation or exceeds the permitted use, you will need to obtain permission directly from the copyright holder. To view a copy of this licence, visit http://creativecommons.org/licenses/by/4.0/.

Open Access funding enabled and organized by Projekt DEAL.

