# Supplementary material for: Serine-dependent redox homeostasis regulates glioblastoma cell survival
Source: Br J Cancer. 2020 Mar 17;122(9):1391–8. doi: 10.1038/s41416-020-0794-x (PMC7188854; doi:10.1038/s41416-020-0794-x)
Supplement: Supplementary file 1 — Supplemental Material [file 41416_2020_794_MOESM1_ESM.pdf]

SUPPLEMENTARY METHODS

Cell lines and culture conditions

LN-229 cells stably expressing a shRNA targeting *HIF-1α, HIF-2 α, HIF-1+2α* and control (*SIMA*, homologue of HIF-1α in drosophila), were kindly provided by C. Depner and T. Acker. Lentiviral production was performed according to the BLOCK-iT™ Lentiviral Pol II miR RNAi Expression System using the plasmid pLenti6/V5-DEST (Invitrogen). Lentiviral infection was carried out using a multiplicity of infection of 60 cells. For selection of polyclonal stable transfected cells 10 µg/mL blasticidin was added to the culture medium.

Suppl. Table 1: Primer pairs for qRT-PCR analysis

	Fwd	Rev
18S	5’-CGGCTACCACATCCAAGGAA-3’	5’-GCTGGAATTACCGCGGCT-3’
SDHA	5’-TGGGAACAAGAGGGCATCTG-3’	5’-CCACCACTGCATCAAATTCATG-3’
PHGDH	5’-CTGCGGAAAGTGCTCATCAGT-3’	5’-TGGCAGAGCGAACAATAAGGC-3’
SHMT1	5’-CTGGCACAACCCCTCAAAGA -3’	5’-AGGCAATCAGCTCCAATCCAA-3’
SHMT2	5’-CCCTTCTGCAACCTCACGAC-3’	5’-TGAGCTTATAGGGCATAGACTCG-3’
HIF-1α	5’-GTCGGACAGCCTCACCAAACAGAGC-3’	5’-GTTAACTTGATCCAAAGCTCTGAG-3’
HIF-2α	5’-GCGCTAGACTCCGAGAACAT-3’	5’-TGGCCACTTACTACCTGACCCT-3’
CAIX	5’-AAGAAGAGGGCTCCCTGAAG-3’	5-‘ TAGCGCCAATGACTCTGGTC-3’
HO-1	5’-CTGCTCAACATCCAGCTCTTTG-3’	5’-AGTGTAAGGACCCATCGGAGA-3’
TXN-1	5’-ACGGTGATGCTGGCAATAGG-3’	5’-CTGGGGTGAGCTCCACCTTA-3’

Amino acid measurements by LC-MS/MS

Amino acid measurements by LC-MS/MS was performed as recently described (1). Cells were lysed using 300 µL of ice-cold 80 % methanol solution and cleared by centrifugation at 13000 rpm for 5 min at 4 °C. 30 µL of sample were mixed with 70 µL of 50 mM TEAB solution and 1 µL of TMT reagent (ThermoFisher Scientific, 0.8 mg TMT reagent resuspended in 41 µL anhydrous acetonitrile). Reactions were performed for 1 hour at room temperature and quenched by addition of

hydroxylamine to a final concentration of 0.5 % and incubation for 15 min. Equal amounts of samples were pooled and diluted 1:100 in 0.1 % formic acid solution for LC-MS analysis.

As an internal standard an analystical standard mix of 17 amino acids (Sigma, catalog no. AAS18-5ML) was diluted 1:250 in water and labeling was performed as described above with TMT-0 reagent. 4 µL of labeled standard was mixed into sample multiplex before dilution.

4 µL of sample was separated on an Easy nLC II (ThermoFisher Scientific) and a 23 cm long, 75 µM ID fused-silica column, which has been packed in house with 3 µM C18 particles (ReproSil-Pur, Dr. Maisch), and kept at 50 °C using an integrated column oven (Sonation). Seperation was performed by a linear gradient from 1.8-85.5 % acetonitrile over 30 min and sprayed directly into an Orbitrap Fusion Lumos mass spectrometer using a nanoFlex ion source (ThermoFisher Scientific, Waltham, MA, USA) at a spray voltage of 2.6 kV. Full scan MS were acquired in range of 295-600 m/z at an orbitrap resolution of 120,000 at m/z 200 with a maximum injection time of 50 ms and an AGC target value of $6x10^5$. Cycle time was set to 1.2 seconds and TMT-0 labelled amino acids were targeted for MS2 scans according to calculated masses. TMT-6 labelled amino acids were selected using a targeted mass difference of 5.0104 Da. To avoid repeated measures dynamic exclusion was set to 5 seconds. Ions were isolated for MS/MS with an isolation window of 0.4 Th, fragmented using high energy collisional dissociation (normalized collision energy of 38) and analysed in the orbitrap with a resolution of 50,000, a maximum injection time of 86 ms and an AGC target value of $5x10^4$.

TMT quantifications were manually extracted from averaged MS2 spectra using Freestyle 1.5 (ThermoFisher Scientific, Waltham, MA, USA).

REFERENCES

1. Murphy JP, Everley RA, Coloff JL, Gygi SP. Combining amine metabolomics and quantitative proteomics of cancer cells using derivatization with isobaric tags. Anal Chem 2014; 86(7):3585–93.

Suppl. Fig. 1

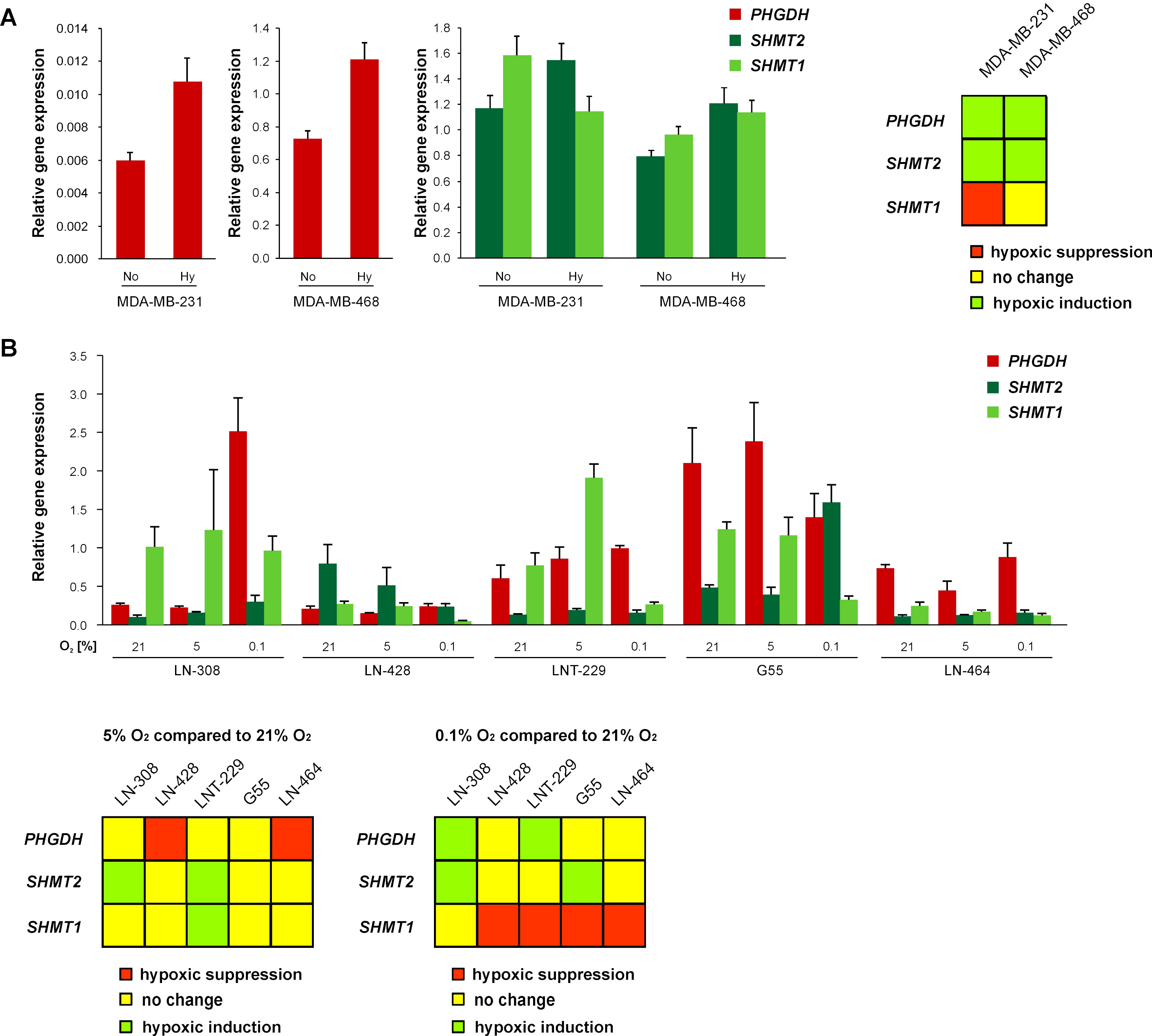

A
Relative gene expression
0.014
0.012
0.010
0.008
0.006
0.004
0.002
0.000
No
Hy
MDA-MB-231
1.4
1.2
1.0
0.8
0.6
0.4
0.2
0.0
MDA-MB-468
1.8
1.6
PHGDH
SHMT2
SHMT1
MDA-MB-231
MDA-MB-468
hypoxic suppression
no change
hypoxic induction
B
3.5
3.0
2.5
2.0
1.5
0.5
O2 [%]
21
5
0.1
LN-308
LN-428
LNT-229
G55
LN-464
5% O2 compared to 21% O2
0.1% O2 compared to 21% O2

Suppl. Fig. 2

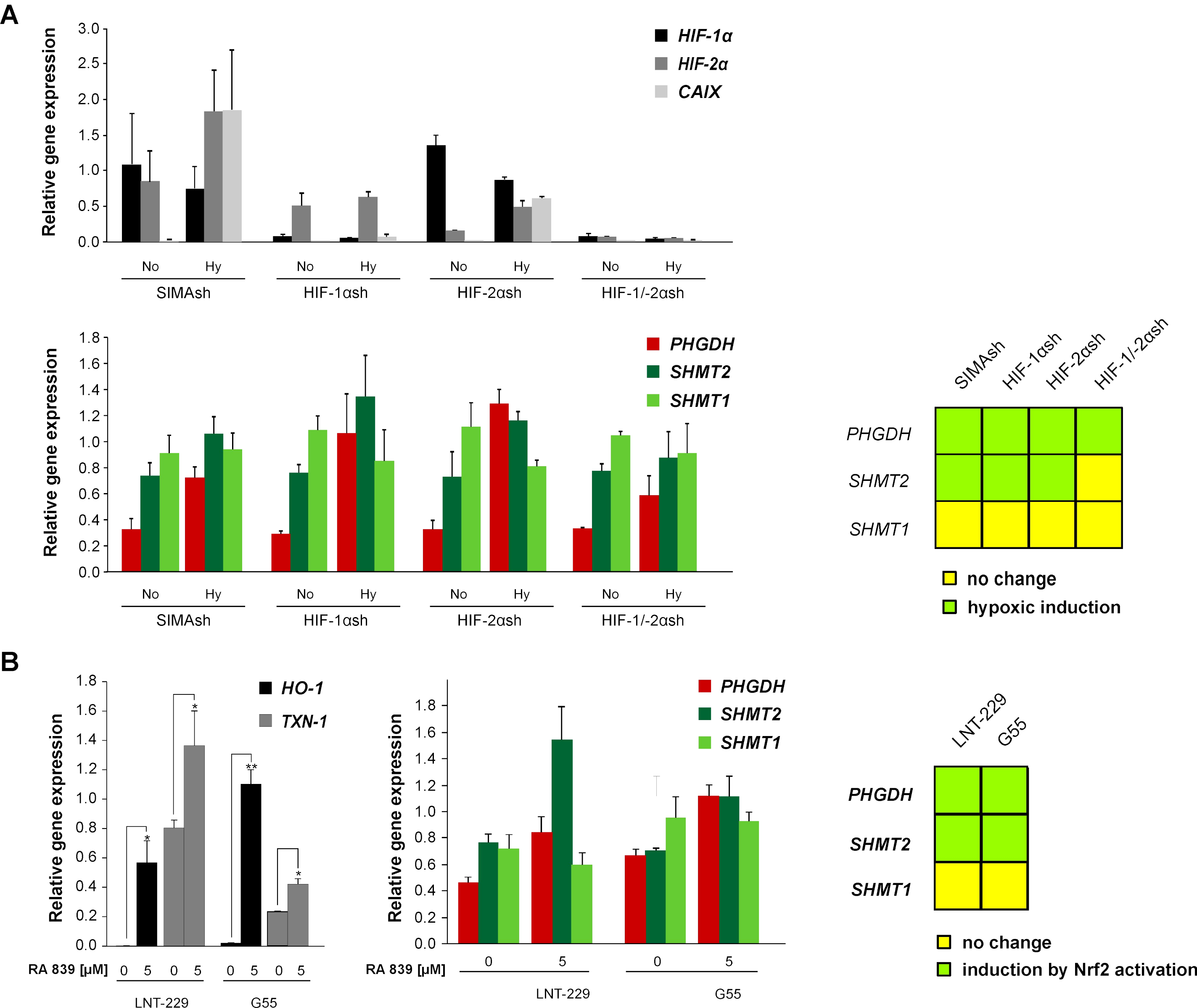
A
Relative gene expression
3.0
2.5
2.0
1.5
1.0
0.5
0.0
HIF-1α
HIF-2α
CAIX
No
Hy
SIMAsh
HIF-1αsh
HIF-2αsh
HIF-1/-2αsh
1.8
1.6
1.4
1.2
0.8
0.6
0.4
0.2
PHGDH
SHMT2
SHMT1
SIMAsh
HIF-1αsh
HIF-2αsh
HIF-1/-2αsh
no change
hypoxic induction
B
HO-1
TXN-1
*
**
RA 839 [µM]
0
5
LNT-229
G55
LNT-229
G55
no change
induction by Nrf2 activation

Suppl. Fig. 3

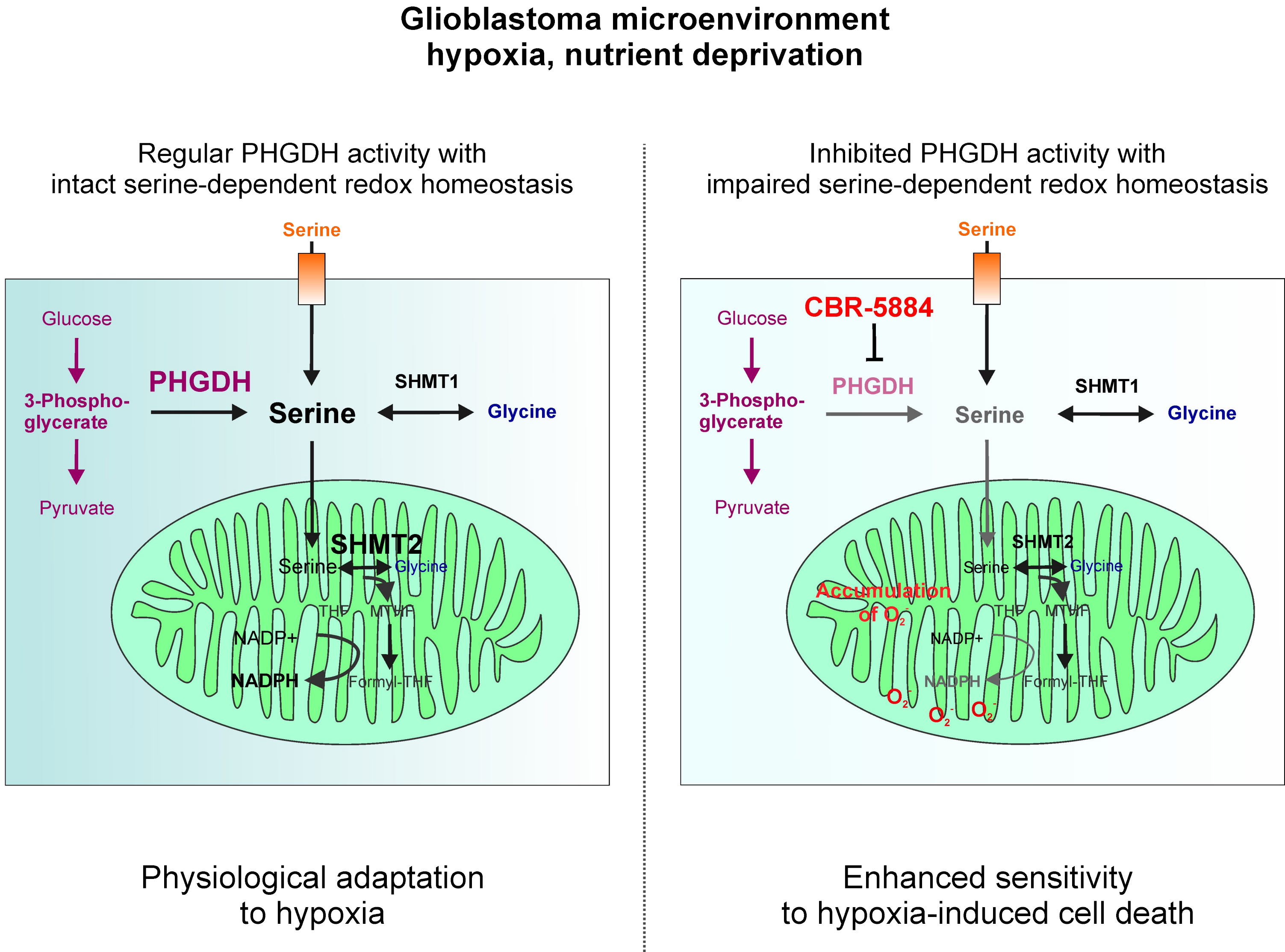
Glioblastoma microenvironment
hypoxia, nutrient deprivation
Regular PHGDH activity with
intact serine-dependent redox homeostasis
Serine
Glucose
3-Phospho-
glycerate
Pyruvate
PHGDH
Serine
SHMT1
Glycine
SHMT2
Serine
Glycine
THF
MTHF
NADP+
NADPH
Formyl-THF
Physiological adaptation
to hypoxia
Inhibited PHGDH activity with
impaired serine-dependent redox homeostasis
Serine
CBR-5884
Glucose
3-Phospho-
glycerate
Pyruvate
PHGDH
Serine
SHMT1
Glycine
SHMT2
Serine
Glycine
Accumulation
of O_2^-
THF
MTHF
NADP+
NADPH
Formyl-THF
O_2^-
O_2^-
O_2^-
Enhanced sensitivity
to hypoxia-induced cell death

Supplementary Figure 1: *PHGDH* and *SHMT2* are upregulated under hypoxic conditions

A, MDA-MB-231 and MDA-MB-468 breast cancer cells were exposed to serum-free DMEM under normoxic or hypoxic (1% oxygen) conditions. Gene expression of the SSP enzymes *PHGDH*, *SHMT1* and *2* was investigated by qPCR. Values are normalized to *18S* as well as *SDHA* housekeeping gene expression (n = 3, mean ± SD). Significant gene induction (*p<0.05 or **p<0.01) is illustrated by green boxes, significant gene suppression (*p<0.05 or **p<0.01) is illustrated by red boxes and no significant change in gene expression is illustrated by yellow boxes. B, glioma cells were exposed to serum-free DMEM under 21, 5 or 0.1% oxygen. Gene expression of the SSP enzymes *PHGDH*, *SHMT1* and *2* was investigated by qPCR. Values are normalized to *18S* as well as *SDHA* housekeeping gene expression (n = 3, mean ± SD). Significant gene induction (*p<0.05 or **p<0.01) is illustrated by green boxes, significant gene suppression (*p<0.05 or **p<0.01) is illustrated by red boxes and no significant change in gene expression is illustrated by yellow boxes.

Supplementary Figure 2: *PHGDH* and *SHMT2* are upregulated under hypoxic conditions in a Nrf2 dependent manner

A, upper panel: LN-229 SIMAsh, HIF-1αsh, HIF-2α and HIF-1/-2α glioma cells were exposed to DMEM containing 10% FCS under normoxic or hypoxic (0.1% oxygen) conditions. Gene expression of *HIF-1α*, *HIF-2α* and the HIF target *carbonic anhydrase IX (CAIX)* was investigated by qPCR. Values are normalized to *18S* as well as *SDHA* housekeeping gene expression (n = 3, mean ± SD). Lower panel: LN-229 SIMAsh, HIF-1αsh, HIF-2α and HIF-1/-2α cells were exposed to serum-free DMEM under normoxic or hypoxic (1% oxygen) conditions. Gene expression of the SSP enzymes *PHGDH*, *SHMT1* and *2* was investigated by qPCR. Values are

normalized to *18S* as well as *SDHA* housekeeping gene expression (n = 3, mean ± SD). Significant gene induction (*p<0.05 or **p<0.01) is illustrated by green boxes and no significant change in gene expression is illustrated by yellow boxes. B, Gene expression of the SSP enzymes *PHGDH*, *SHMT1* and *2* as well as *HO-1* and *TXN-1* in LNT-229 and G55 cells was investigated by qPCR under normoxic conditions in serum-free DMEM with or without 5 μM of the Nrf2 activator RA 839 for 6 h. Values are normalized to *18S* as well as *SDHA* housekeeping gene expression (n = 3, mean ± SD, n.s. = not significant, *p<0.05, **p<0.01). For SSP enzymes significant gene induction (*p<0.05 or **p<0.01) is illustrated by green boxes and no significant induction is illustrated by yellow boxes.

Supplementary Figure 3: Schematic overview of metabolic changes in PHGDH impaired glioma cells

The PHGDH inhibitor CBR-5884 or *PHGDH* gene-suppression reduce intracellular serine levels in human GB cells (right panel). A consequential decrease in the NADPH/NADP+ ratio with elevated ROS levels ultimately triggers enhanced sensitivity to hypoxia-induced cell death.
